# Selective Lithium–Magnesium Separation from Brines by Ionic-Liquid Solvent Extraction: Molecular Mechanisms, Extractant Design, and Process Optimization

**DOI:** 10.3390/ijms27209005

**Published:** 2026-10-11

**Authors:** Mauricio Mura, Jonathan Castillo, Edelmira Gálvez, Williams Leiva, Pía Hernández, Felipe M. Galleguillos-Madrid, Rossana Sepúlveda, Norman Toro

**Affiliations:** 1 Departamento de Ingeniería Química y Procesos de Minerales, Facultad de Ingeniería, Universidad de Antofagasta, Antofagasta 1240000, Chile; mauricio.mura@alumnos.ucn.cl (M.M.); pia.hernandez@uantof.cl (P.H.); 2 Departamento de Ingeniería en Metalurgia, Universidad de Atacama, Copiapó 1531772, Chile; rossana.sepulveda@uda.cl; 3 Departamento de Ingeniería Metalúrgica y Minas, Universidad Católica del Norte, Antofagasta 1270709, Chile; egalvez@ucn.cl; 4 Facultad de Ingeniería, Universidad San Sebastián, Sede Concepción, Concepción 4030000, Chile; williams.leiva@uss.cl; 5 Centro de Desarrollo Energético Antofagasta, Universidad de Antofagasta, Antofagasta 1240000, Chile; felipe.galleguillos.madrid@uantof.cl; 6 Faculty of Engineering and Architecture, Universidad Arturo Prat, Avenida Arturo Prat 2120, Iquique 1110939, Chile

**Keywords:** direct lithium extraction (DLE), lithium purification, high Mg/Li ratio, magnesium removal, liquid–liquid extraction, alternative solvents, ion coordination chemistry, quantum chemical calculations

## Abstract

The growing global demand for lithium (Li^+^), driven by its strategic role in energy storage technologies, faces a critical challenge in its recovery from brines: the selective separation of Li^+^ and magnesium (Mg^2+^) ions. Because Li^+^ (ionic radius ≈ 76 pm) and Mg^2+^ (≈ 72 pm) have almost identical ionic radii despite their different charge, their coordination and hydration behaviour are difficult to discriminate at the molecular level. The high Mg^2+^ content in natural brines, such as those from the Atacama Salar, delays the efficiency of conventional extraction processes, which also result in significant Li^+^ losses. In this context ionic liquids (ILs) are emerging as alternative solvents with tunable properties, low volatility, and high thermal stability, although their environmental performance depends strongly on their chemical structure, toxicity, aqueous solubility, and recyclability, offering advantages over traditional organic extractants. This study evaluates the use of ionic liquids in liquid–liquid extraction systems for the selective removal of Mg^2+^, analysing operational parameters such as pH, organic-to-aqueous phase ratio (O/A), and extractant concentration. This review critically compares ionic-liquid-based extraction systems by relating Mg^2+^/Li^+^ selectivity to extractant structure, coordination chemistry, and key operational variables, while also considering the main limitations associated with their application to high-salinity brines. By linking macroscopic extraction performance to the underlying coordination chemistry, hydration-shell disruption, and computational screening tools such as quantum chemical calculations and COSMO-RS, this approach supports the rational, molecular-level design of environmentally responsible extractants for brines with a high Mg/Li ratio.

## 1. Introduction

Lithium resources occur naturally in two forms: as minerals and as liquids. The limited availability of lithium in mineral deposits, together with the high extraction costs, has steered the industry towards its liquid-state resources, which are estimated to contain more than 85% of the world’s recoverable lithium [1]. Lithium has been classified as one of the critical or near-critical elements in several recent studies, primarily due to its importance and high demand in green technologies, as well as its market availability [2]. Furthermore, it is regarded as a strategic resource that has been widely applied in various fields, particularly in lithium-ion batteries used for vehicles and energy storage [3].

Lithium resources are found mainly in hard rocks and saline lake brines, the latter accounting for more than 70% of the world’s lithium reserves [4]. Table 1 shows various locations around the world that contain different types of brines, along with the concentrations of their main ions. Chile possesses the most significant lithium deposit on the planet: the Salar de Atacama. Covering an area of more than 2800 km^2^ between the Andes and Domeyko mountain ranges in the Antofagasta Region, it hosts the highest known average concentration of lithium [5].

Currently, Chile has become a major producer due to the vast size of the Salar de Atacama and the high quality of its lithium brines, resulting in the country producing 23% of the world’s lithium in 2019 [2]. Neighbouring countries such as Argentina and Bolivia also contain large salt flats; however, they have lower lithium concentrations and higher levels of contaminants, such as magnesium, which in many cases render their economic exploitation unfeasible. In fact, although Bolivia and Argentina possess larger lithium resources than Chile, the economically recoverable potential is considerably lower [6].

Brine is one of the most important potential resources for lithium recovery, primarily using evaporative methods. Conventional processes involve drilling the ground to extract the brine using pumps, depositing the brine in evaporation ponds to remove water and concentrate the salts. The main impurities are then removed through fractional crystallisation, followed by the final step of refining the lithium salts in a chemical plant [7]. Lithium-containing brine resources can be categorised into three types: evaporative, geothermal, and oilfield brines [8,9]. This phenomenon has been attributed to the retention of lithium by the precipitated salts. The residual brine is highly enriched with Mg^2+^ compared to Na^+^ and K^+^, making lithium recovery from this residual brine particularly difficult [10,11].

**Table 1:** Chemical composition of various brines [10].

Source	(wt.%)							
	Na^+^	K^+^	B^3+^	Li^+^	Mg^2+^	Ca^2+^	Cl^−^	SO_4_^2−^
Salar de Atacama, Chile	9.1	2.62	0.04	0.183	0.965	0.038	18.95	2.00
HombreMuerto, Argentina	9.9–10.30	0.24–0.97	-	0.068–0.121	0.018–0.14	0.019–0.09	15.80–16.80	0.53–1.14
Salar de Uyuni, Bolivia	7.06	1.17	0.071	0.0321	0.65	0.0306	5	-
Searles Lake, USA	11.08	2.53	-	0.0054	-	0.0016	12.3	4.61
Great Salt Lake, USA	3.70–8.70	0.26–0.72	0.007	0.0018	0.5–0.97	0.026–0.036	7.00–15.60	0.94–2.00
Taijinaier, China	5.63	0.44	-	0.031	2.02	0.02	13.42	3.41

At present, the main challenge in the extraction of Li^+^ from brines lies in the efficient and selective separation of Li^+^/Mg^2+^. Magnesium ions (Mg^2+^) are always found together with lithium ions (Li^+^) in brines, often in greater quantities, and must be separated in an efficient and cost-effective manner [12,13]. Magnesium poses a major obstacle to lithium extraction because these two elements occupy diagonal positions in the periodic table, resulting in several chemical similarities, including their relatively small ionic radii. However, important differences exist in the aqueous solubility of their corresponding compounds: lithium hydroxide and, to a lesser extent, lithium carbonate are appreciably more soluble in water than the corresponding magnesium compounds. Quantitatively, the six-coordinate ionic radii of Li^+^ (≈76 pm) and Mg^2+^ (≈72 pm) differ by less than 6%, yet their hydration enthalpies diverge sharply (approximately −520 kJ mol^−1^ for Li^+^ versus approximately −1900 kJ mol^−1^ for Mg^2+^) because of the higher charge density of the divalent cation. This combination of near-identical size but markedly different hydration free energy is the molecular root of the Li^+^/Mg^2+^ separation problem, since any extractant able to accommodate one cation in its inner coordination sphere will, to some extent, also accommodate the other. Furthermore, the Mg^2+^/Li^+^ ratios are relatively high in most Chinese brines and in some North American brines, making it a global challenge to selectively extract Li^+^ from brines with high Mg^2+^/Li^+^ ratios [14,15,16]

Since the separation of lithium and magnesium represents the main challenge in lithium extraction, the conventional or traditional process consists of several stages. Initially, multi-stage solar evaporation is employed until a concentrated brine is obtained, typically containing between 5% and 6% Li^+^, respectively [17]. Subsequently, this brine is transferred to a processing plant where a liquid–liquid extraction process is carried out to remove boron [18]. In the next step, the Mg^2+^ and Ca^2+^ contents are separated through precipitation and solid–liquid separation, using lime and Na_2_CO_3_. However, these purification stages result in Li+ losses, as a portion becomes trapped during the precipitation processes.

In recent decades, several methods have been developed to extract Li+ from brines, such as solvent extraction [19], precipitation [20], adsorption [21], membrane separation [22], and electrochemical methods [23,24,25,26,27,28]. Among these, solvent extraction is the most promising for industrial applications due to its high Li^+^ extraction efficiency, high Li^+^/Mg^2+^ separation selectivity, simple operation, and low cost [29,30]. These advantages are most directly established for conventional molecular extractants; ionic-liquid-based variants introduce additional practical considerations, including extractant cost, viscosity, and regeneration requirements, that must be weighed before their industrial feasibility can be assumed on the same basis. Moreover, solvent extraction offers notable advantages, including greater selectivity in metal extraction and the ability to operate under moderate reaction conditions. However, constant control of the solution pH is required [31].

The present study proposes the development of a selective extraction system for Mg^2+^ from brines using ionic liquids as extractant agents. This approach addresses the challenge posed by the high concentration of Mg^2+^ in lithium-bearing brines, which constitutes a major barrier to the efficient recovery of Li^+^. The use of ionic liquids, often described as green solvents due to their low volatility and high chemical and thermal stability, has been proposed as a more environmentally friendly alternative to conventional organic extractants [32]. However, this classification is not universal, and the practical and environmental performance of a given ionic liquid depends on its specific cation–anion combination, viscosity, phase-separation behaviour, and ease of recovery.

**Figure 1:**
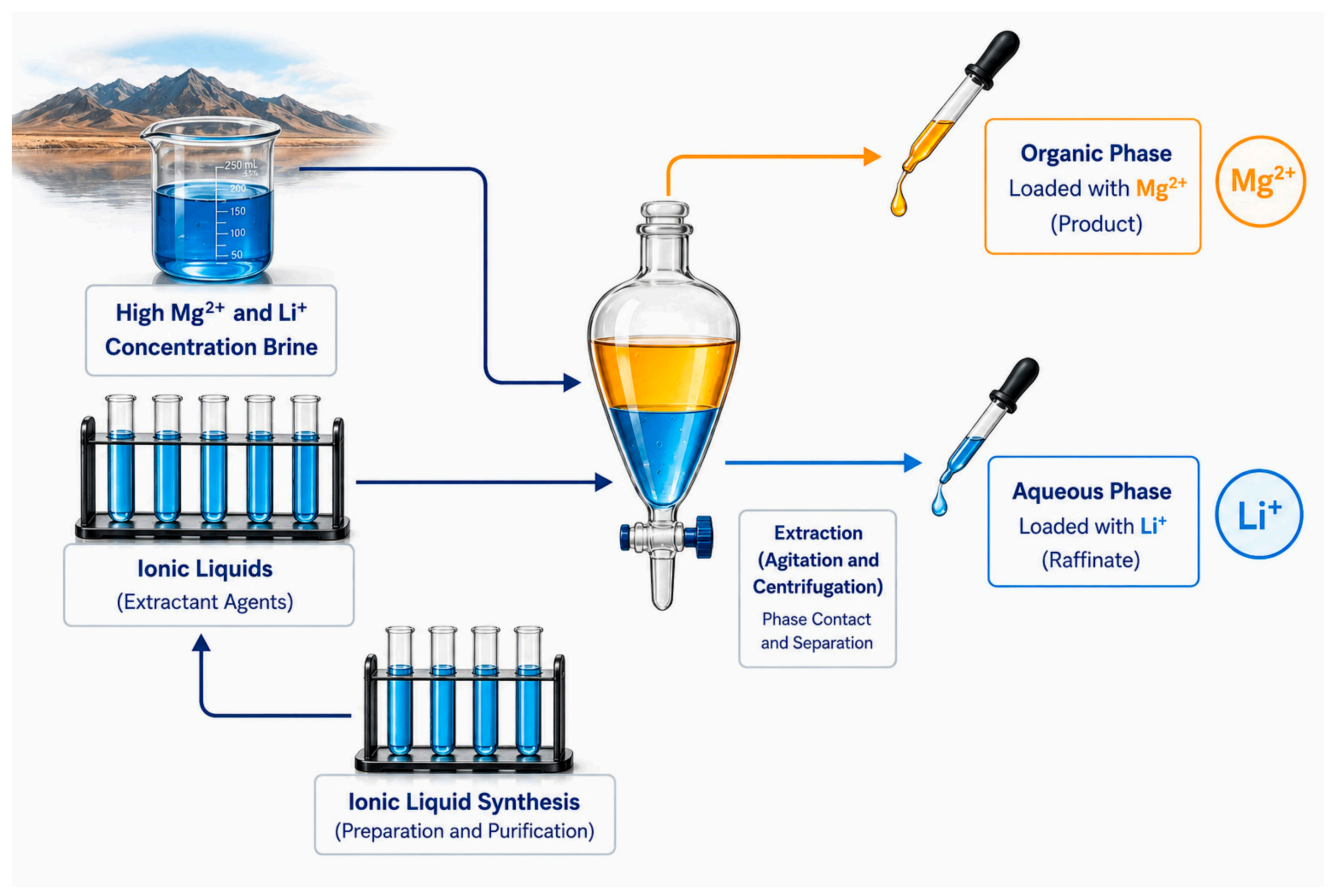
Flow diagram of solvent extraction for Mg^2+^ removal using ionic liquids.

Figure 1 schematically illustrates the solvent extraction process for the selective removal of magnesium (Mg^2+^) from high-salinity brines using ionic liquids as extractants. Initially, the ionic liquid is synthesized and subsequently employed as the organic phase. The organic phase is then contacted with the brine during a liquid–liquid extraction step, where Mg^2+^ is selectively transferred from the aqueous phase to the ionic liquid through cation-exchange and complexation mechanisms, while Li^+^ remains predominantly in the aqueous phase. Following phase separation, two distinct phases are obtained: an organic phase consisting of the ionic liquid loaded with Mg^2+^, and an aqueous raffinate enriched in Li^+^ while containing significantly lower concentrations of Mg^2+^. This selective extraction enhances the Mg/Li separation, facilitating the downstream processing of lithium-rich brines for lithium recovery.

Despite growing interest in ionic liquids for lithium recovery, few studies have systematically evaluated the effect of operational parameters—such as O/A ratio, pH, and extractant composition—on Mg/Li selectivity under realistic brine conditions. This study addresses this gap by correlating these variables with extraction performance, providing insights that can guide solvent design and process optimisation for industrial-scale lithium recovery [33].

Recent reviews have examined lithium recovery from brines from complementary perspectives, including solvent-extraction chemistry, lithium coordination and chelator design, ionic-liquid-based extraction systems, and broader comparisons among emerging separation technologies. In particular, Jiang et al. [34] comprehensively reviewed the use of ionic liquids as diluents, extractants, and co-extractants for lithium solvent extraction, including their extraction mechanisms, operating conditions, challenges, and future prospects. However, the present review adopts a different organizing perspective by focusing specifically on Li^+^/Mg^2+^ selectivity in brines with high Mg/Li ratios, with particular emphasis on Mg^2+^-selective purification while minimizing Li^+^ co-extraction. Rather than considering molecular chemistry and process operation separately, this review connects ion hydration and dehydration, coordination chemistry, ion–extractant interactions, and ionic-liquid structure with key operational variables such as pH, organic-to-aqueous phase ratio, and ionic-liquid concentration. Furthermore, Mg^2+^ extraction and Li^+^ co-extraction are comparatively assessed to establish structure–selectivity–process relationships that may guide the rational design and optimization of ionic-liquid extraction systems for high-Mg/Li brines.

Accordingly, the main objective of this review is to critically assess ionic-liquid-based solvent extraction systems for Li^+^/Mg^2+^ separation and to establish relationships between molecular-level selectivity, extractant design, and key operational variables such as pH, organic-to-aqueous phase ratio, and ionic-liquid concentration. Particular attention is given to Mg^2+^-selective purification strategies that minimize Li^+^ co-extraction in brines with high Mg/Li ratios.

The literature search was conducted using Scopus, Web of Science, ScienceDirect, and Google Scholar. The main search terms included combinations of “lithium–magnesium separation”, “Mg/Li separation”, “high Mg/Li brine”, “ionic liquid extraction”, “solvent extraction”, “magnesium removal”, “lithium extraction”, and “ionic liquids”. The search focused primarily on peer-reviewed publications published between 2000 and 2026. Earlier studies were also included when they provided fundamental information on solvent-extraction mechanisms, metal coordination, or established concepts relevant to Li^+^/Mg^2+^ separation. Priority was given to studies reporting brine composition, extraction performance, selectivity, operating conditions, and molecular or thermodynamic aspects of the separation process. Studies not directly related to brine purification or Li^+^/Mg^2+^ separation were excluded from the detailed analysis.

## 2. Fundamentals

### 2.1. Conventional Solvent Extraction Mechanisms and Governing Equations

Solvent extraction has been presented as a potential alternative for application in the lithium industry, particularly for complex brines with low lithium content and a high Mg/Li ratio [35], as well as for the recovery of valuable elements from lithium batteries [36,37,38]. Common extractants for Li^+^ include amide extractants [39], diketone extractants [40], and neutral phosphate extractants [41], among others [41].

Neutral phosphate extractants have shown the most promising prospects for research and development, becoming a major focus in lithium extraction from Salt Lake brines both domestically and internationally. As a representative of this class of extractants, tributyl phosphate (TBP) demonstrates excellent performance in lithium extraction from brines with high magnesium content.

Solvent extraction technology exhibits clear advantages, including high selectivity for Li^+^, attractive economic feasibility, and the ability to operate continuously at an industrial scale. These are widely cited potential advantages of solvent extraction in general; realising them in practice, particularly for ionic-liquid-based systems, additionally requires favourable viscosity, efficient phase separation, acceptable mass-transfer rates, and economical extractant regeneration. Numerous extraction systems involving tributyl phosphate (TBP) have been used for Li^+^ extraction from brines with a high Mg/Li ratio [42,43,44,45], and the mechanism and principle of the extraction process can be described by the following general equations (1, 2, and 3) for Li^+^ extraction in the presence of Mg^2+^ [46].

The following reactions are presented as overall stoichiometric representations of the extraction process. Unless otherwise stated, the species shown should not be interpreted as unique molecular species experimentally demonstrated under all brine conditions.

Extraction step.

Li^+^(aq) + Fe^3+^(aq) + 4Cl^−^(aq) ↔ LiFeCl_4_(aq) (1)

LiFeCl_4_(aq) + *n*S (org) ↔ LiFeCl_4_∙*n*S (org) (2)

Re-Extraction step.

LiFeCl_4_∙*n*S (org) + HCl ↔ HFeCl_4_∙*n*S (org) + LiCl (aq) (3)

In chloride-rich media, Fe^3+^ forms chloro-complexes such as FeCl_4_^−^. The extraction of Li^+^ with neutral extractants such as TBP can therefore be described in terms of ion association between Li^+^, FeCl_4_^−^, and the extractant molecules present in the organic phase. In this context, the notation LiFeCl_4_ and LiFeCl_4_·nS used in Equations (1)–(3) represents an overall stoichiometric association rather than a unique molecular species necessarily present in the aqueous or organic phase [46].

Shi et al. [47] proposed improving the Li^+^ extraction capacity of TBP-based systems by incorporating an additional extractant [48]. In their study, they determined that the addition of a second extractant (TBP–kerosene) allows adjustment of the proportion of (FeCl_4_·nTBP)^3^, also known as a complex ionic liquid, which enhances both the operability and efficiency of the Li^+^ extraction process in the presence of high Mg^2+^ concentrations. Therefore, in this approach, FeCl_4_ must be added to the organic phase to form the complex (FeCl_4_·nTBP)^3^. The mechanism and principles of this Li^+^ extraction process can be represented by the following equations (Equations (4, 5 and 6)):

(4) $${\mathrm{Mg}}^{2+}\left(\mathrm{aq}\right)+{2\mathrm{Fe}}^{3+}\left(\mathrm{aq}\right)+8{\mathrm{Cl}}^{-}\left(\mathrm{aq}\right)↔{\mathrm{Mg}(\mathrm{Fe}{\mathrm{Cl}}_{4})}_{2}(\mathrm{aq})$$

(5) Mg(FeCl4)2aq+nS Org↔Mg(FeCl4)2·nS (Org)

(6) 2Li+aq+Mg(FeCl4)2·nS org↔2LiFeCl4·nS org+MgCl2(aq)

In these equations, S represents the extractant P248. In chloride-rich media, Fe^3+^ forms chloro-complexes such as FeCl_4_^−^, while acidic conditions are maintained to limit Fe^3+^ hydrolysis. For the Mg^2+^-containing system, charge balance requires the association of one Mg^2+^ ion with two FeCl_4_^−^ anions. Accordingly, Equation (4) has been corrected to preserve both stoichiometry and charge balance. The notation Mg(FeCl_4_)_2_ is used here as a simplified representation of the Mg^2+^/FeCl_4_^−^ ion association. Subsequent coordination with P248 and the proposed exchange of Mg^2+^ by Li^+^ are therefore presented as schematic steps of the extraction mechanism rather than as evidence for a unique molecular species under concentrated brine conditions.

Therefore, the Li^+^ extraction capacity and phase separation performance in kerosene P248 were related to the P248 concentration, Fe/Li molar ratio, phase ratio, and water acidity. As a representative, tributyl phosphate (TBP) shows excellent performance in lithium extraction from brines with high magnesium content [19,23].

Currently, several studies have been developed focusing on the use of different extractants or molecular extractants aimed at removing magnesium present in brine ponds, due to its high concentration, rather than focusing on lithium recovery. Table 2 presents a compilation of studies in which various extractants were used for magnesium removal from lithium brines, achieving in some cases extraction efficiencies exceeding 80% for magnesium.

In addition, the general equation (Equation (7)) describing the magnesium extraction process using molecular extractants is presented.

2Na A (org) + Mg^+2^ (aq) ⇆ MgA_2_ (org) + 2Na^+^ (aq) (7)

The general equation shown describes the mechanism of magnesium (Mg^2+^) extraction from an aqueous phase into an organic phase using molecular extractants in the form of a sodium salt (NaA). In this process, two molecules of the extractant (NaA) present in the organic phase react with a Mg^2+^ ion dissolved in the aqueous phase, forming a neutral complex (MgA_2_) that transfers to the organic phase, while Na^+^ are released into the aqueous phase to maintain charge balance. This reaction is reversible, and its efficiency depends on the equilibrium between the phases, being representative of systems in which magnesium is exchanged for light cations (such as Na^+^) through complexation with organic extractants.

Solvent extraction for Mg/Li separation has been under continuous investigation due to the inherent difficulty of achieving selective separation of these two elements. To improve process efficiency and enhance separation selectivity, it has been essential to test mixtures of extractants to maximise the individual effects of each component. Thus, Zhang et al. [53] improved system performance by combining the extractants trioctylamine (N235) and bis(2-ethylhexyl) phosphate (D2EHPA) in TBP. In this way, the researchers achieved Mg^2+^ extraction efficiencies of up to 71% and a Li^+^/Mg^2+^ selectivity of 30.86 [54]. Because this factor is defined as β(Li/Mg) = D_Li_/D_Mg_, its value of 30.86 numerically favours Li^+^ over Mg^2+^; re-expressed in the Mg^2+^-removal-oriented form relevant to this system’s stated purpose, β(Mg/Li) = 1/30.86 ≈ 0.03, indicating that the N235/D2EHPA/TBP combination itself provides only modest intrinsic Mg^2+^-over-Li^+^ discrimination, and that the 71% Mg^2+^ extraction efficiency obtained here is better attributed to favourable mass-action conditions (high O/A ratio and Mg/Li ratio in the feed brine) than to a high separation factor in the Mg-removal sense.

### 2.2. Ionic Liquids as Alternative Extractants

Ionic liquids (ILs) are salts with melting points typically below 100 °C that have attracted considerable attention as alternative solvents and extractants in catalysis [55], synthesis [23], electrochemistry [56], and separation processes [57]. Their low volatility, low flammability, high thermal stability, and tunable physicochemical properties can provide important operational advantages over conventional volatile organic solvents [58]. However, these properties alone do not necessarily imply a lower environmental impact. The environmental performance of an ionic liquid strongly depends on the chemical nature of its cation and anion, as well as on its toxicity, biodegradability, water solubility, persistence, and potential release during operation. In liquid–liquid extraction, partial solubility of the ionic liquid in the aqueous phase may result in solvent losses and contamination of the raffinate, making recovery and regeneration important considerations for process design. In addition, some fluorinated ionic-liquid anions require particular attention because of concerns related to their persistence, hydrolytic stability, and potentially hazardous degradation products. Therefore, ionic liquids should not be universally classified as “green” solvents; rather, their environmental performance should be evaluated on a case-by-case basis considering their composition, recovery, reuse, and life-cycle behaviour [59,60,61].

Ionic liquids are generally composed of asymmetric cations and anions and are commonly described as tunable “designer solvents”, since their cationic and anionic components can be individually modified to adjust physicochemical and extraction properties [62,63]. This tunability has motivated the use of predictive computational screening approaches, such as the conductor-like screening model for real solvents (COSMO-RS), to pre-select cation–anion combinations for selective Li^+^ extraction before laboratory synthesis, thereby reducing the trial-and-error burden traditionally associated with ionic-liquid development [64].

Mixtures composed of two (or more) extractants are used to improve extraction and separation properties compared to the use of each extractant individually, a phenomenon known as synergistic extraction. Many mixed systems have been investigated for the extraction and separation of metal ions [58]. Recently, mixed extraction systems combining ionic liquids and molecular extractants (IL–ME systems) have attracted attention due to their green characteristics, extraction and separation efficiency, synergistic effect, and cost reduction [65,66,67].

While the use of ionic liquids can effectively enhance the physicochemical properties of organic phases, previous studies have mainly focused on the extraction performance of ionic liquids, which have been used primarily as extractants or synergistic co-extractants [68]. The advantages of ionic liquids have also suggested their use as a reaction medium that is, as an organic solvent to improve the separation of magnesium from lithium using organic extractants [69].

During extraction, the cations or anions present in ionic liquids may transfer to the aqueous phase when the reaction occurs via an ion-exchange or synthesis mechanism [70,71]. This phenomenon can create challenges in regenerating systems that employ ionic liquids. On the other hand, the use of ionic liquids as substitutes for the extractant medium (ME) in solvent extraction systems also increases the chemical complexity of the process. In this context, phosphonium-based ionic liquids, dialkyl phosphate ionic liquids, and binary or ternary combinations have been studied, showing favourable synergistic or sintering effects in their interaction with other extractants [23,58]. Common ionic liquids can also be prepared in the laboratory by mixing Aliquat 336 with organophosphorus acids such as Cyanex 272. Studies have demonstrated the excellent efficiency of ionic liquids derived from Aliquat 336 and Cyanex 272 in solvent extraction systems [32,72,73]

There are also studies proposing the use of ionic liquids in direct lithium extraction (DLE). For instance, Shi et al. successfully extracted lithium using [N_4444_][DEHP] and [N_8888_][DEHP], although some difficulties were observed during the lithium stripping process [74,75,76].

In the following study, Sun et al. [68] synthesised the ionic liquid [A336][P204] by mixing the corresponding components. The organic extraction phase consisted of this ionic liquid as the extractant, dissolved in an organic diluent (MIBK), with the proportion of ionic liquid varied between 30% and 70%. The study was conducted using a synthetic brine simulating the residual solution from a salt lake after potassium extraction, containing 50.42 g/L Mg^2+^. Under the investigated conditions, the [A336][P204]/MIBK system showed preferential Mg^2+^ extraction while limiting Li^+^ co-extraction, demonstrating its potential for selective Mg^2+^ removal from concentrated lithium-bearing brines [77].

Furthermore, the author proposes a mechanism for the extraction process, which is expressed by Equation (8) [68].

Mg^2+^ (Aq) + 2Cl^−^ (Aq) + 2 [A336][P204] (Org) → MgCl_2_∙2 [A336][P204] (Org) (8)

Equation (8) represents the overall stoichiometric association proposed for Mg^2+^ extraction with [A336][P204]. In chloride-rich brines, Mg^2+^ remains strongly solvated and may interact with chloride depending on the solution composition and ionic strength. Therefore, the representation MgCl_2_·2[A336][P204] should be interpreted as a proposed extracted stoichiometry rather than as evidence that undissociated MgCl_2_ is the predominant aqueous species or that a single molecular complex exclusively describes the organic-phase speciation.

Li et al. [78] proposed an eco-friendly solvent extraction method using binary extractants (ionic liquids) to effectively separate lithium and magnesium. They prepared a binary extractant composed of Aliquat 336 and Versatic Acid 10, referred to as [A336][V10]. The authors evaluated its capacity to extract magnesium from both a synthetic concentrated brine containing 106 g/L Mg and 10 g/L Li, and a synthetic original brine with 15 g/L Mg, 80 g/L Na, and 0.2 g/L Li. Through a series of batch counter-current experiments and mixer-settler tests, they found that [A336][V10] could completely remove magnesium from the original brine in three continuous counter-current extraction stages, with only about 10% co-extraction of lithium. The loaded organic phase could then be stripped and regenerated using water. The entire process (extraction and stripping) required no acids or bases, relying solely on the difference in chloride concentration during both stages. This method offers a more sustainable and environmentally friendly alternative to current state-of-the-art processes, representing a step forward in the sustainable production of Li_2_CO_3_ from brines [78]. The authors also propose a mechanism for the extraction process, which is shown below.

m[A336][V10]_org_ + MCl_m(aq)_ ↔ M[V10]_org_ + m[A336]Cl_(org)_ (9)

where “MCl_m_” represents the salts that are extracted and m = 2 for magnesium extraction.

The extraction of Mg^2+^ ions in IL based media occurs primarily through an ion-pair formation mechanism involving coordination between Mg^2+^ and the anionic component of the ionic liquid, typically a phosphinic or phosphonic acid anion. For example, in the system [A336][P204], the extraction reaction can be expressed as:

MgCl2 (aq) + 2[A336][P204](org) ⇌ MgCl_2_∙2[A336][P204](org) (10)

Equation (10) provides a simplified representation of the association between MgCl_2_ and [A336][P204] in the organic phase. This expression is consistent with the overall stoichiometric formulation presented in Equation (8) and should be interpreted as an alternative schematic representation of the extracted Mg-containing association rather than as evidence for a distinct molecular species.

The extraction selectivity depends strongly on both anion and cation structures in the IL. The length of the alkyl chains in the quaternary ammonium cation ([A336]^+^, [N8888]^+^, etc.) controls phase polarity and viscosity, which in turn affect mass transfer and complex stability. Shorter chains typically enhance phase miscibility and facilitate faster ion exchange, while longer chains increase hydrophobicity and extraction selectivity [79,80,81,82]

From a thermodynamic standpoint, the observed Mg/Li selectivity correlates with the Gibbs free energy of solvation (ΔG_sol_ᵥ) and the stability constants of the extracted complexes. Kinetic factors—such as diffusion across the organic–aqueous interface and interfacial mass-transfer coefficients—also play an important role in determining the overall extraction efficiency. Systems with lower viscosity and better interfacial activity generally display higher Mg^2+^ transfer rates [69,83,84].

Despite these advantages, the relatively high cost of ionic liquids remains an important limitation for their implementation in large-scale solvent extraction processes. The economic feasibility of IL-based systems depends not only on extraction efficiency and selectivity, but also on the cost and availability of the starting materials, the number of synthesis and purification steps, solvent losses, and the possibility of recovering and reusing the ionic liquid over multiple extraction–stripping cycles. In this regard, structurally simpler ionic liquids prepared from readily available precursors, particularly some ammonium-, phosphonium-, and protic-based systems, may offer greater potential for large-scale applications than more complex task-specific or fluorinated ionic liquids. Moreover, using ILs as extractants or synergistic components at relatively low concentrations, rather than as the bulk solvent phase, can reduce the required IL inventory and consequently the associated process cost. Therefore, future scale-up of IL-based Mg^2+^/Li^+^ separation should consider extraction performance together with solvent recovery, recyclability, synthesis complexity, and make-up requirements. High IL recovery and reuse over multiple cycles will be particularly important for offsetting their higher initial cost and improving their competitiveness with conventional solvent-extraction systems [85].

### 2.3. Quantitative Discussion and Selectivity

To quantitatively evaluate the extraction efficiency and selectivity of the ionic liquid-based system, the classical parameters of solvent extraction were applied: the distribution coefficient (D), the separation factor (β), and the extraction efficiency (E%). These parameters enable the comparison of different systems under similar experimental conditions shown in Equation (11) [69,86].

(11) $$D_{i}=\frac{C_{i,\mathrm{org}}}{C_{i,\mathrm{aq}}}$$

where C_i_,org and C_i_,aq correspond to the concentrations of ion in the organic and aqueous phases, respectively, after extraction equilibrium is reached. The extraction efficiency is calculated in Equation (12).

(12) $$eE_{i}\left(\%\right)=\frac{D_{i}}{D_{i}+\frac{V_{\mathrm{aq}}}{V_{\mathrm{org}}}}\cdot 100$$

while the selectivity between lithium and magnesium is expressed by the separation factor (β) in Equation (13).

(13) $$\beta_{\text{Li/Mg}}=\frac{D_{\mathrm{Li}}}{D_{\mathrm{Mg}}}$$

High β values indicate a greater preference of the extractant system for Li^+^ over Mg^2+^, making this parameter a key criterion for selecting the appropriate ionic liquid and optimizing operational conditions [86].

Because β(Li/Mg) is defined with D_Li_ in the numerator, it is the natural metric for processes whose explicit objective is Li^+^ extraction into the organic phase (direct lithium extraction, DLE-type processes), where a large β(Li/Mg) value signals a strong Li^+^-over-Mg^2+^ preference. For processes whose objective is instead Mg^2+^ removal, that is, selectively transferring Mg^2+^ to the organic phase while retaining Li^+^ in the aqueous raffinate, the complementary separation factor β(Mg/Li) = D_Mg_/D_Li_ = 1/β(Li/Mg) is the more directly interpretable quantity, since a large β(Mg/Li) value (equivalently, a small β(Li/Mg), well below 1) indicates good Mg^2+^-selective performance. Both quantities describe the same underlying equilibrium and are related by simple inversion, so no information is lost by reporting either one; the choice is a matter of which process objective the value is meant to communicate. This review surveys both Mg^2+^-removal systems, where Mg^2+^ is the targeted species and Li^+^ co-extraction (or Li^+^ loss) is the quantity to be minimised, and direct Li^+^-extraction systems, where Li^+^ is the targeted species and Mg^2+^ co-extraction is the quantity to be minimised. Because several of the cited studies report their results exclusively as β(Li/Mg), this value is retained when discussing them to remain faithful to the original data, with the complementary β(Mg/Li) value given alongside it whenever the system under discussion is better understood in Mg^2+^-removal terms. Throughout the text, the terms ‘Mg^2+^ removal’ and ‘Li^+^ extraction’ are therefore used deliberately to identify which of the two cations is the targeted transfer species in each study, since the same ionic-liquid platform can in some cases be operated as either, or even combine both objectives in sequential process stages.

This can be validated through the wide variability of these parameters, which depend on the nature of the ionic liquid, the diluent phase, and the system conditions (pH, O/A ratio, and initial concentration). The authors Zhou et al. [53] studied the extraction of Li^+^ and Mg^2+^ from brines with a high Mg/Li ratio using the [A336][P204] + TBP system, achieving a lithium extraction efficiency of 94.2% and a separation factor β ≈ 539 at pH = 6 and O/A = 1. This is a Li^+^-extraction-type result (β(Li/Mg) ≫ 1, equivalently β(Mg/Li) ≈ 1/539 ≈ 0.002), consistent with the high reported Li^+^ extraction efficiency rather than with an Mg^2+^-removal objective. Other recent studies, such as Zhang et al. [54], demonstrated that structural modification of the ionic liquid, for example, increasing the alkyl chain length or using hydrophobic anions like [NTf_2_]^−^, enhances both the Li distribution coefficient (D_li_) and the stability of the extracted complex, thereby reducing Mg^2+^ co-extraction and improving the overall process efficiency.

### 2.4. Molecular-Level and Computational Insights into Ion Selectivity

Beyond Mg^2+^-removal strategies, a complementary line of research has pursued the direct, highly selective extraction of Li^+^ itself using ionic-liquid-based synergistic extractants, leaving Mg^2+^ preferentially in the raffinate. Working with the system [HOEMIM][Tf_2_N] combined with trioctyl phosphate (TOP), Yu et al. [23] reported a single-stage Li^+^ extraction efficiency of 83.16%, together with a separation factor β(Li/Mg) of 742.11, one of the highest values reported to date for a synergistic ionic-liquid system. This is also a Li^+^-extraction-mode result (β(Mg/Li) ≈ 1/742.11 ≈ 1.3 × 10^−3^), reported in the β(Li/Mg) convention common to direct-lithium-extraction studies. Complementary molecular-level studies combining ionic liquids with tributyl phosphate under different solvent environments have further clarified how electrostatic potential and non-covalent interactions among the ionic liquid, Li^+^, and TBP govern extraction selectivity, providing a mechanistic basis for the rational design of next-generation extractants [87]. These findings indicate that, alongside Mg^2+^-selective removal, direct Li^+^-selective extraction constitutes a viable and increasingly competitive route for brines with a high Mg/Li ratio, broadening the toolbox available for industrial process design.

**Figure 2:**
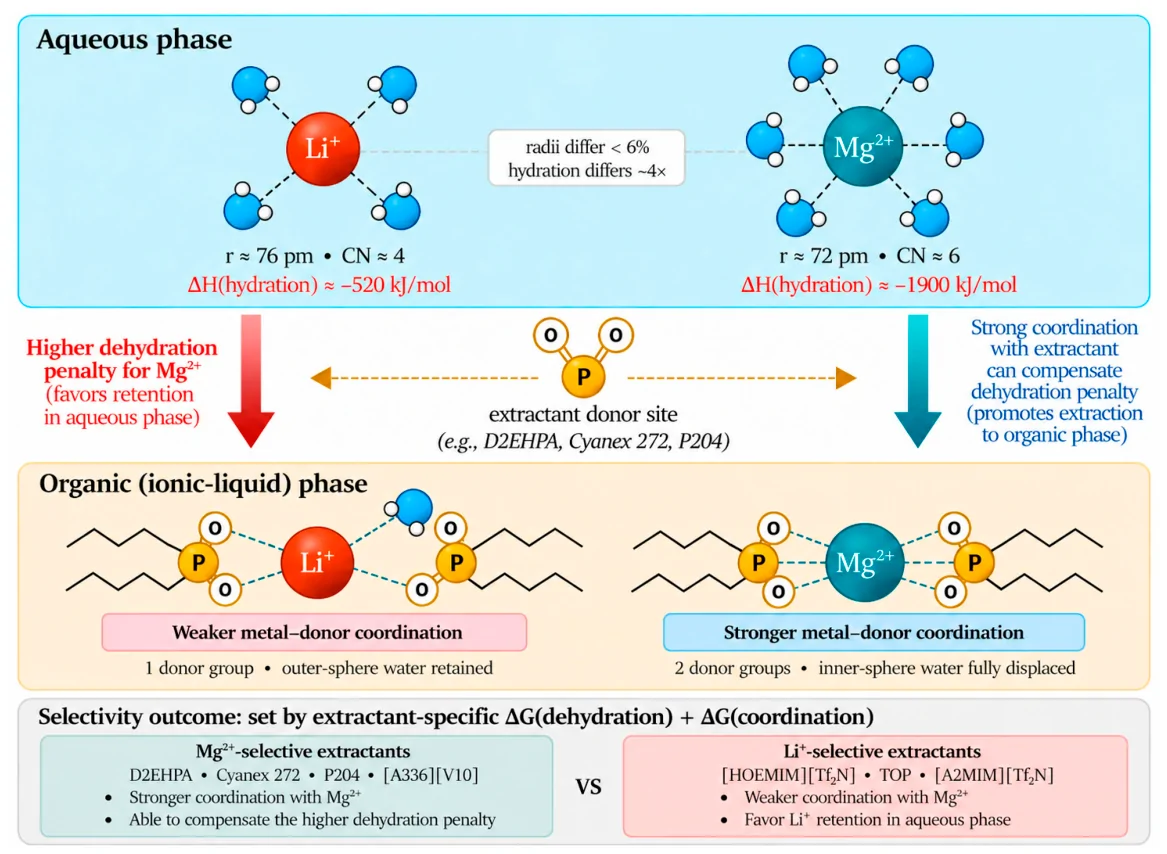
Molecular origin of Li^+^/Mg^2+^ selectivity. Although Li^+^ and Mg^2+^ have similar ionic radii, Mg^2+^ is much more strongly hydrated and therefore requires a larger energetic penalty for dehydration and transfer from the aqueous phase. This effect alone favors Mg^2+^ retention in the aqueous phase. However, sufficiently strong coordination of Mg^2+^ with suitable donor groups in the extractant can compensate for this dehydration penalty, so the final Li^+^/Mg^2+^ selectivity results from the balance between dehydration and extractant–ion coordination.

Figure 2 summarises this competition. At the molecular level, extraction selectivity ultimately reflects a competition between the energy required to strip part of the hydration shell from each cation and the energy gained upon forming a new coordination bond with the extractant. Because Mg^2+^ is much more strongly hydrated than Li^+^, its transfer from the aqueous phase requires a substantially larger dehydration penalty. Therefore, considering dehydration alone, Mg^2+^ would be preferentially retained in the aqueous phase. However, the overall extraction selectivity is determined by the balance between this dehydration penalty and the stabilization gained upon coordination with the extractant. Consequently, sufficiently strong and selective interactions between Mg^2+^ and suitable donor groups can compensate for its higher dehydration cost and promote its transfer to the organic phase. Thermodynamic frameworks that explicitly couple this molecular picture to macroscopic phase behaviour, such as the ePC-SAFT equation of state applied to organic–inorganic complex electrolyte systems, have reproduced experimental Li^+^ extraction data while providing a quantitative link between ion solvation and bulk extraction efficiency [87]. In parallel, quantum chemical descriptors, including electrostatic potential maps and non-covalent interaction indices, have been used to visualise how specific donor groups on the extractant compete with water molecules for the cation’s coordination sphere, offering a mechanistic explanation for why small changes in cation or anion structure can produce large changes in Mg^2+^/Li^+^ selectivity [87]. Predictive screening tools such as COSMO-RS extend this molecular reasoning to the extractant design stage itself, allowing candidate cation–anion pairs to be ranked by their calculated affinity for Li^+^ over Mg^2+^ before any synthesis is undertaken [64]. Together, these computational and thermodynamic approaches indicate that further gains in Li^+^/Mg^2+^ selectivity are more likely to come from molecular-scale engineering of the extractant’s coordination environment than from further empirical optimisation of process variables alone.

More specifically, the Mg^2+^/Li^+^ selectivity differences compiled above can be rationalised, at least qualitatively, from the donor-atom chemistry and denticity of the extractant functional group rather than from the ionic-liquid cation alone. Mg^2+^ has a charge density (z^2^/r) roughly four times that of Li^+^ despite their similar ionic radii, which makes it a harder, more polarising Lewis acid with a strong preference for hard, electronegative oxygen donors and for chelating (multidentate) ligands able to form stable five-membered rings around the cation. Acidic organophosphorus anions such as D2EHPA, Cyanex 272, [P204]^−^, and [P507]^−^ present two P=O/P-O^−^ oxygen donors per deprotonated molecule and typically coordinate Mg^2+^ as a 2:1 (anion:metal) chelate, which matches this hard, high-charge-density cation well and underlies their consistently high Mg^2+^ selectivity. β-Diketone anions (HPMBP, HTTA, HDBM) behave similarly once deprotonated, forming bidentate O,O-chelates whose effective acidity, and therefore the pH at which Mg^2+^ binding becomes favourable, is controlled by the aryl/alkyl substituents on the diketone backbone; the comparatively higher acidity of HPMBP relative to HDBM is consistent with its lower onset pH for Mg^2+^ extraction among the three β-diketone systems compared here.

By contrast, systems that achieve high Li^+^ selectivity typically pair a neutral, monodentate phosphoryl donor (TBP or TOP) with a bulky, weakly coordinating anion such as [NTf_2_]^−^/[Tf_2_N]^−^ or [BPh_4_]^−^. These anions carry a delocalised negative charge and low basicity, so they do not compete with the extractant for coordination of the cation and do not favour chelate formation; instead, they stabilise the extracted species mainly through ion-pairing and electrostatic lattice-type interactions, which scale less unfavourably with the lower charge density of Li^+^ than with that of Mg^2+^. The neutral P=O donor of TBP/TOP can complete the coordination sphere of the smaller, singly charged Li^+^ without the steric or electronic penalty that a comparable interaction with the more strongly hydrated Mg^2+^ would incur, consistent with the markedly higher β(Li/Mg) values reported for the [HOEMIM][Tf_2_N]+TOP and [Bmim][BPh_4_]+TBP systems relative to the phosphinic/phosphonic- and β-diketone-based Mg^2+^-removal systems. The quaternary ammonium or imidazolium cation of the ionic liquid ([A336]^+^, [N1444]^+^, [N4444]^+^, [N8888]^+^, [Bmim]^+^) plays a comparatively indirect role in most of these mechanisms: because it typically pairs with the acidic or weakly coordinating anion rather than binding the target cation directly, its alkyl chain length mainly tunes the hydrophobicity, viscosity, and aggregation state of the organic phase rather than the intrinsic Mg^2+^/Li^+^ donor preference, consistent with extraction magnitude, rather than selectivity itself, varying with alkyl chain length among the [A336]^+^, [N1444]^+^, [N4444]^+^, and [N8888]^+^ cations paired with these anions. Carboxylate-type anions such as Versatic Acid 10 ([V10]^−^) occupy an intermediate position: their single, more sterically hindered oxygen donor provides weaker-field, lower-denticity coordination than the phosphinic/phosphonic anions, consistent with the different Mg/Li selectivity ratios observed for [A336][V10] relative to [A336][P204]-type systems. Taken together, these structure-selectivity relationships indicate that donor-atom hardness and denticity, rather than the ionic-liquid cation identity by itself, are the primary molecular levers governing whether a given ionic-liquid system behaves as an Mg^2+^-removal or a Li^+^-extraction platform.

## 3. Operational Parameters

The operational variables discussed below (pH, the organic-to-aqueous phase ratio, and ionic liquid concentration) are macroscopic levers acting on the underlying dehydration and coordination equilibria that govern Li^+^/Mg^2+^ selectivity. Solution pH governs the protonation state of acidic extractants and, by extension, the availability of anionic donor sites able to compete with water for the cation’s coordination sphere; the organic-to-aqueous ratio and extractant concentration shift the mass-action balance between the hydrated aqueous cation and its dehydrated, extractant-bound counterpart. Interpreting the trends reported in the following subsections through this molecular lens helps explain why apparently similar extractant systems can show markedly different Mg^2+^/Li^+^ selectivity under different operating conditions.

### 3.1. Experimental Studies

Zhang et al. [54] investigated Mg^2+^ extraction from a high-Mg/Li brine containing 54.04 g/L Mg^2+^ and 3.22 g/L Li^+^, corresponding to an Mg/Li mass ratio of approximately 16.8. Sixteen functional ionic liquids were synthesized by combining four quaternary ammonium cations ([A336]^+^, [N1444]^+^, [N4444]^+^, and [N8888]^+^) with four saponified anions ([C272]^−^, [P204]^−^, [P507]^−^, and [V10]^−^). Because of the relatively high viscosity of the ionic liquids, different diluents were evaluated to improve mass transfer and phase separation, with MIBK providing favorable extraction behaviour. Among the systems tested, an organic phase containing 50 vol% [A336][V10] and 50 vol% MIBK showed the best overall performance under the investigated conditions. At an O/A ratio of 6:1, Mg^2+^ extraction reached 60.98%, while Li^+^ co-extraction remained at 7.15%. The authors also reported that the ionic-liquid cation structure influenced extraction performance, with shorter alkyl-chain cations generally showing higher extraction rates within the investigated series. In addition to extraction selectivity, electrical conductivity was considered because the study coupled Mg^2+^ extraction with subsequent electrodeposition of metallic magnesium.

The extraction efficiencies summarized in Table 3 should be interpreted within the specific experimental conditions of each study rather than as directly comparable performance indicators. Higher O/A ratios and extractant concentrations generally increase the amount of extractant available relative to the aqueous metal loading, which can enhance Mg^2+^ transfer to the organic phase. Likewise, pH strongly influences the protonation state and coordination ability of acidic extractants, thereby modifying Mg^2+^/Li^+^ selectivity. Diluent properties, particularly polarity and viscosity, can also affect complex stability, mass transfer, and phase disengagement. In addition, multistage extraction can produce substantially higher cumulative removal efficiencies than single-stage batch experiments.

Li and Binnemans [51] evaluated Mg^2+^ extraction using C923/IL and TBP/IL systems containing [HOEmin][NTf_2_]. Importantly, these experiments were performed using a dilute model solution containing only 0.10 M MgCl_2_, rather than a concentrated high-salinity brine. Under these conditions, the C923/IL system achieved quantitative Mg^2+^ extraction at an IL concentration of 0.20 M, whereas the TBP/IL system reached approximately 75%. Although these results demonstrate the strong Mg^2+^ affinity of the C923/IL system, the reported 100% extraction should not be interpreted as directly equivalent to performance in concentrated brines. In comparison, systems such as [A336][P204] and [A336][V10] were evaluated with synthetic brines containing approximately 50–54 g/L Mg^2+^, providing conditions that are more representative of high-Mg/Li brines.

Unlike the Mg^2+^-removal-type systems discussed above, the following example illustrates a direct Li^+^-extraction-type process, in which Li^+^ is the targeted transfer species and Mg^2+^ is subsequently rejected in a separate purification step. In the subsequent study, conducted by R. Li et al. [79], the ionic liquid 1-butyl-3-methylimidazolium tetraphenylborate ([Bmim][Bph_4_]) was synthesised and used as a co-extractant together with tributyl phosphate (TBP) and bromochloromethane (CH_2_BrCl) as extractant and diluent, respectively. The research achieved a Li+ extraction efficiency of 99.47% after four stages, using an organic/aqueous ratio (O/A) of 1, a molar concentration of 2.6 M, and a TBP volume fraction of 50%. Unlike other studies, this work focused on Mg^2+^ removal during the washing stage. For this purpose, the organic phase was treated with a mixed NaCl and LiCl solution, achieving a magnesium removal efficiency of nearly 100% without significant lithium loss (0%). In the stripping stage, a Na_2_CO_3_ solution was used as the re-extractant, achieving total lithium recovery (100%). Finally, ten complete extraction cycles were performed, demonstrating that the organic phase exhibited excellent reusability and stability throughout the process.

**Figure 3:**
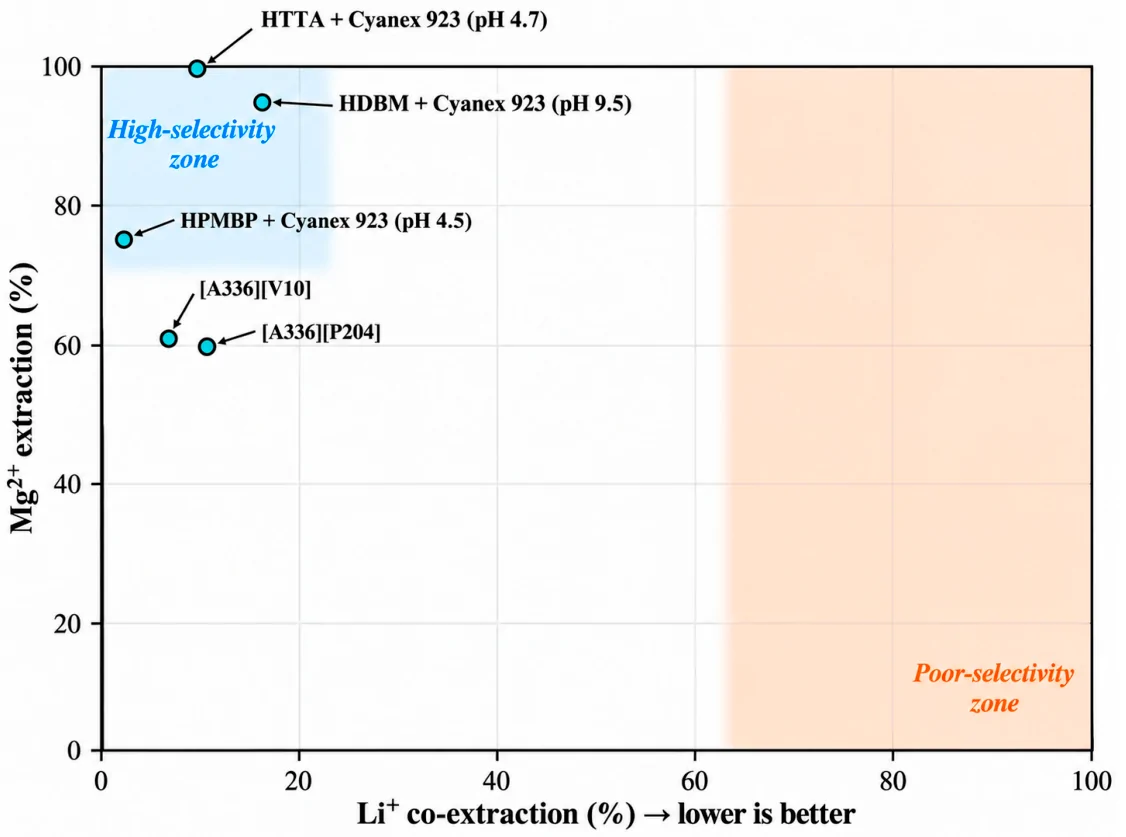
Selectivity map of the extraction systems reported in Table 3, comparing Mg^2+^ extraction with Li^+^ co-extraction. Systems located toward the upper-left region combine high Mg^2+^ extraction with low Li^+^ co-extraction, indicating favourable performance for selective Mg^2+^ removal.

Figure 3 consolidates the Mg^2+^/Li^+^ performance of the systems compiled in Table 3, mapping extraction against co-extraction for each.

### 3.2. Effect of the O/A Phase Ratio on Extraction and Washing Performance

Given the high concentration of Mg^2+^ in synthetic brines, it is necessary to optimise the conditions of the liquid–liquid extraction process to achieve efficient separation between this ion and Li^+^. One of the most critical parameters is the organic-to-aqueous phase ratio (O/A), which has a direct impact on the efficiency of magnesium extraction. Experimental results show that increasing the O/A ratio significantly improves the Mg^2+^ extraction rate. This is attributed to the greater volume of the organic phase available to interact with the metal ions in solution, thereby promoting the transfer of magnesium from the aqueous to the organic phase [54].

In this regard, Sun et al. [68] demonstrated that the O/A phase ratio strongly influences Mg^2+^ extraction. Increasing the O/A ratio to 10:1 resulted in an Mg^2+^ extraction efficiency of 83.99%. Under the optimized conditions of 60% [A336][P204] and 40% MIBK at an O/A ratio of 6:1, the system achieved 59.72% Mg^2+^ extraction with only 10.70% Li^+^ co-extraction. These results demonstrate that both the phase ratio and the organic-phase composition significantly affect Mg^2+^ extraction and Li^+^ co-extraction.

A different role of the O/A ratio was reported by R. Li et al. [79] during the washing stage of a Li^+^-extraction process based on [Bmim][Bph_4_]. In this case, the objective was not the direct extraction of Mg^2+^ from the feed brine, but the removal of co-extracted impurities from the loaded organic phase while minimizing Li^+^ loss. The authors observed that the washing efficiencies of Mg^2+^, K^+^, and Li^+^ varied with the phase ratio, indicating that an appropriate O/A ratio is required to remove Mg^2+^ without significantly washing lithium from the organic phase.

An O/A ratio of 6:1 was found to provide an optimal balance between both objectives, achieving a Mg^2+^ washing efficiency close to 100% and virtually no lithium loss (0%). This finding is potentially relevant for industrial applications, pending confirmation at larger scale, as it enables the design of more efficient purification processes by reducing the magnesium content in the organic stream prior to the final lithium stripping stage. This, in turn, contributes to improving the purity of the final product and the overall efficiency of the system.

Overall, these studies demonstrate that the O/A ratio is an important variable in both extraction and washing operations, but its optimum value is system- and stage-dependent. Therefore, comparisons of O/A effects should consider the extraction system, feed composition, process objective, and whether the reported value corresponds to primary extraction or a subsequent washing step. This assessment is based on bench-scale extraction and washing data; confirming economic viability at an industrial scale will additionally require evaluating ionic-liquid cost, viscosity, phase-separation behaviour, and regeneration performance over extended operating cycles.

### 3.3. pH Effect

Li and Binnemans [51] examined the effect of pH on the selective extraction of Mg^2+^ and Li^+^ using three synergistic β-diketone/Cyanex 923 systems: HPMBP, HTTA, and HDBM. In the case of the HPMBP system, the results showed that at a pH close to 3.3, a Mg^2+^ extraction efficiency of approximately 75% was achieved, which increased to quantitative Mg^2+^ extraction when the pH increased above approximately 4.5. Under these conditions, Li^+^ extraction was negligible, reflecting a high selectivity towards Mg^2+^ in acidic media. However, as the pH progressively increased above 4, Li^+^ co-extraction increased, reaching a plateau of approximately 85% at pH values above 6.7. At pH 4.5, Li^+^ extraction was only 2.3%, whereas Mg^2+^ extraction was quantitative, confirming the strong influence of pH in discriminating between the two cations. These results are summarized in Table 3.

**Figure 4:**
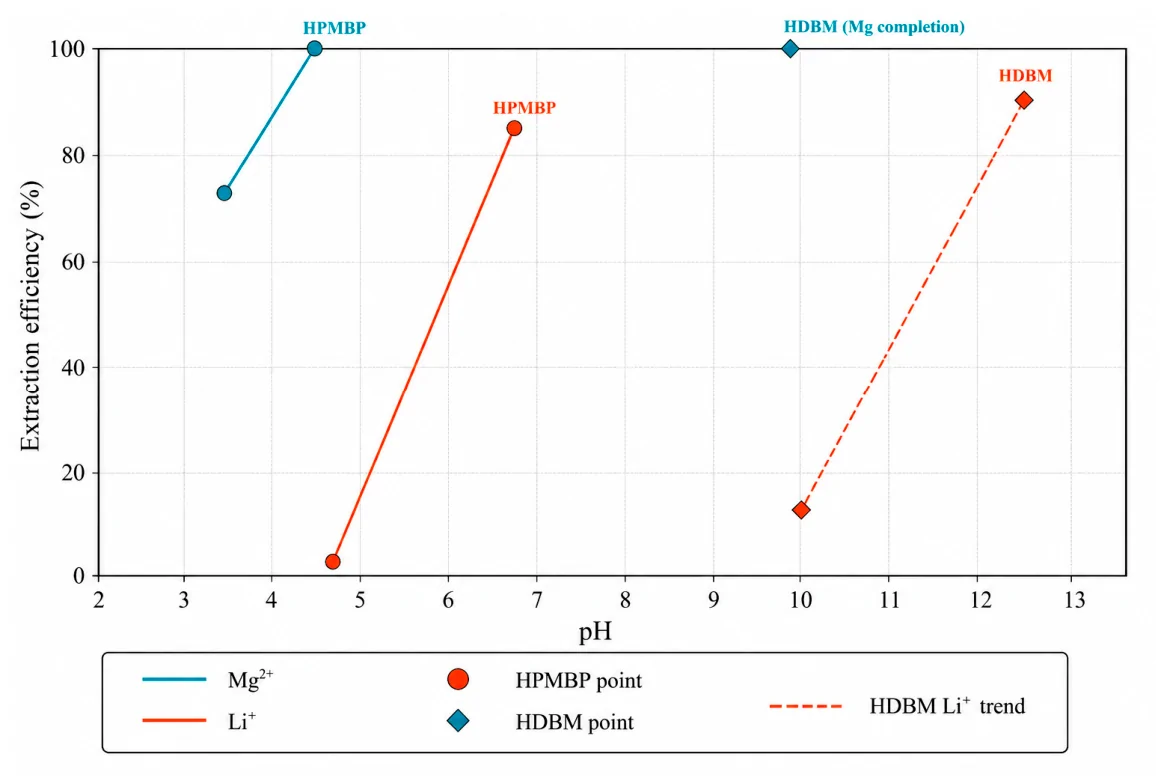
Effect of pH on Mg^2+^ and Li^+^ extraction for the HPMBP and HDBM synergistic β-diketone systems. Markers represent literature-reported values [51], and lines are guides for the eye. For both extractants, Mg^2+^ extraction increases at lower pH than Li^+^ extraction, indicating a pH window favorable for selective Mg^2+^ removal.

In the HTTA system, the Mg^2+^ extraction trend was similar to that observed with HPMBP, although with slight differences in the equilibrium isotherm. In this case, Li^+^ co-extraction remained low at pH values below 4.5, increasing sharply once this threshold was exceeded. At higher pH values, Li^+^ co-extraction increased sharply, reaching approximately 98% at pH 8.5. This behaviour indicates a progressive loss of Mg^2+^/Li^+^ selectivity as the pH increases. By contrast, the HDBM system displayed a markedly different behaviour, as Mg^2+^ extraction was not completed until pH values approached 9.5, at which point Li^+^ co-extraction reached approximately 16% (Table 3), higher than that recorded in the other two systems. At still higher pH values (around 11.8), Li^+^ co-extraction increased to approximately 92%, confirming that the selectivity toward Mg^2+^ decreases substantially under strongly alkaline conditions. The pH-dependent extraction behaviour of the HPMBP and HDBM systems is illustrated in Figure 4 [88].

On the other hand, the study conducted by Sun et al. [68], using ionic liquids of the type [A336][P204], revealed a different behaviour regarding the pH effect. The authors observed that changes in the brine pH had minimal influence on extraction efficiency, suggesting that the organic phase composed of ionic liquids exhibits high resistance to acidic conditions and can maintain performance over a wide operational pH range (1 to 7). Under these conditions, Li^+^ co-extraction in a single stage remained consistently low, not exceeding 14% under the investigated conditions. In contrast, Mg^2+^ extraction showed an increasing trend, reaching values above 40% as the composition of the organic phase was optimised.

These studies suggest that some ionic-liquid-based extraction systems can exhibit comparatively low pH sensitivity within specific operating ranges. However, this behaviour depends strongly on the ionic-liquid composition, extractant chemistry, and brine matrix and should therefore not be generalized to all IL-based extraction systems [89]. Similar behaviour was reported by Zhou et al. [85], who observe srd relatively limited variation in Mg^2+^ removal with pH, with the highest extraction values obtained around pH 6–7.

### 3.4. Effect of Ionic Liquid Concentration

Li and Binnemans [13] investigated the effect of [HOEmin][NTf_2_] concentration on Mg^2+^ extraction using C923/IL and TBP/IL systems. As summarized in Table 3, the C923/IL system showed higher Mg^2+^ extraction than the corresponding TBP/IL system under the investigated conditions. At an [HOEmin][NTf_2_] concentration of 0.20 M, the C923/IL system achieved quantitative Mg^2+^ extraction, whereas the TBP/IL system reached approximately 75% Mg^2+^ extraction under the reported experimental conditions. At this concentration, magnesium extraction was practically quantitative. A notable aspect of this system’s behaviour was the molar ratio between the added ionic liquid and the extracted magnesium. The results showed an Ionic Liquid/Magnesium ratio of approximately 2.1–2.2, the reported IL/Mg molar ratio of approximately 2.1–2.2 is consistent with an overall stoichiometry involving approximately two [NTf_2_]^−^ anions per Mg^2+^ ion, in accordance with charge-balance considerations. However, in the TBP/IL system, although an increase in extraction efficiency was also observed with increasing [HOEmin][NTf_2_] concentration, the relationship was not linear, and the overall extraction efficiency was considerably lower than that observed with C923. This indicates that the synergy between C923 and the IL is more favourable for Mg^2+^ extraction, and that the ionic liquid concentration plays a critical role in determining process performance.

In a different system, R. Li et al. [79] investigated the influence of the [Bmim][BPh_4_]/Li^+^ molar ratio on Li^+^ extraction. In this case, the process objective differs from that of the [HOEmin][NTf_2_]-based Mg^2+^-removal systems discussed above, since [Bmim][BPh_4_] was employed primarily for selective Li^+^ extraction followed by impurity removal during the washing stage. Increasing the IL/Li molar ratio enhanced Li^+^ extraction up to an optimum ratio of approximately 2.6, beyond which further increases produced little additional improvement. Under the optimized conditions, high Li^+^ extraction was achieved during the extraction stage, while Mg^2+^ was subsequently removed during the washing stage with negligible Li^+^ loss. These results demonstrate that the IL/Li molar ratio is an important parameter for controlling extraction performance and selectivity in this system.

### 3.5. Critical Assessment and Industrial Applicability

From a process-design perspective, the most promising ionic-liquid architectures are not necessarily those providing the highest extraction efficiency under simplified laboratory conditions, but those combining high Mg^2+^/Li^+^ selectivity with low viscosity, limited aqueous-phase loss, efficient phase separation, and effective regeneration. ILs used as extractants or synergistic components in liquid–liquid extraction offer high molecular tunability and can potentially be integrated into conventional solvent-extraction circuits. However, their relatively high viscosity, solvent inventory, possible transfer to the aqueous phase, and regeneration requirements remain important limitations for large-scale implementation. In addition to viscosity and regeneration, mass-transfer rates across the organic–aqueous interface and the long-term chemical and thermal stability of the ionic liquid itself under repeated extraction–stripping cycles, where exposure to concentrated brine, acidic or alkaline stripping agents, and elevated temperature can promote hydrolysis or decomposition of the cation or anion, are additional factors that determine whether a given system remains economically attractive over its operating lifetime. Supported ionic-liquid membranes can reduce IL consumption and enable continuous extraction, although long-term membrane stability and IL loss remain important challenges. Compared with adsorption and membrane-based technologies, IL solvent extraction may be particularly attractive when high throughput and compatibility with existing hydrometallurgical equipment are required, whereas adsorption and membrane processes can reduce the need for bulk organic-phase handling.

Industrial relevance also depends strongly on the composition of the brine used for validation. Results obtained using realistic high-salinity and multicomponent brines, containing competing ions such as Na^+^, K^+^, Ca^2+^, and Cl^−^ and representative Mg/Li ratios, are more informative for scale-up than results obtained from simplified binary solutions. High extraction efficiencies measured under idealized conditions should therefore be interpreted cautiously, because competitive complexation, viscosity, mass-transfer limitations, phase disengagement, and IL losses may become more significant in concentrated brines. Future studies should consequently prioritize realistic brine matrices, multicycle regeneration, solvent-loss measurements, and continuous or multistage operation to establish the practical viability of IL-based Li^+^/Mg^2+^ separation.

## 4. Conclusions

The molecular-level analysis presented above indicates that Li^+^/Mg^2+^ selectivity in ionic-liquid extraction systems is governed primarily by the balance between the cation dehydration energy and the strength of the coordination interactions formed with the extractant, rather than by bulk phase properties alone. Ionic-liquid-based solvent extraction represents a potentially promising alternative for Li^+^/Mg^2+^ separation because ILs offer low volatility, generally high thermal and chemical stability, and tunable cation–anion combinations that can be tailored to improve process selectivity. Realising this potential at an industrial scale is nevertheless contingent on resolving the ionic-liquid cost, viscosity, phase-separation, mass-transfer, and regeneration limitations discussed in this review. However, these characteristics do not inherently guarantee environmental sustainability. The environmental performance of IL-based extraction systems also depends on factors such as toxicity, biodegradability, aqueous solubility, solvent losses, persistence, regeneration efficiency, and the chemical nature of the constituent ions.

Synergistic ionic-liquid systems have demonstrated high selectivity and efficiency for Mg^2+^ removal. Experimental studies show that specific combinations of ILs with diluents or co-extractants can achieve Mg^2+^ extraction efficiencies above 80–90% under particular experimental conditions, while limiting Li^+^ co-extraction. Nevertheless, these values should be interpreted considering differences in brine composition, Mg/Li ratio, extractant concentration, diluent, pH, O/A ratio, and extraction configuration among the reported studies.

In parallel, several ionic-liquid platforms have been developed for the complementary objective of direct Li^+^ extraction, achieving separation factors β(Li/Mg) substantially greater than unity. Because this review addresses both process objectives, separation-factor values are reported in whichever orientation, β(Li/Mg) or its reciprocal β(Mg/Li) = 1/β(Li/Mg), is natural to the stated objective of the system being discussed, rather than defaulting to a single convention regardless of context. Readers comparing separation-factor magnitudes across studies should therefore first confirm whether each study targets Mg^2+^ removal or Li^+^ extraction, since the two objectives call for opposite orientations of the same underlying distribution-coefficient ratio.

The adjustment of operational parameters remains essential for optimizing extraction performance. The O/A phase ratio, pH, and ionic-liquid concentration directly influence both Mg^2+^ extraction and Li^+^ co-extraction. Increasing the O/A ratio generally enhances Mg^2+^ transfer to the organic phase, whereas pH strongly affects metal coordination and selectivity. Therefore, optimization must balance high Mg^2+^ removal with minimal Li^+^ loss rather than considering extraction efficiency alone.

Future development of IL-based extraction processes should therefore integrate molecular selectivity with solvent recovery, regeneration, reuse, aqueous-phase losses, and environmental assessment. Validation using realistic multicomponent brines and multicycle or continuous operation will also be necessary to establish their practical applicability. In this context, coupling computational screening tools such as COSMO-RS with experimental validation may accelerate the rational design of extractants tailored to specific brine chemistries [64], while multifunctional extraction systems may offer opportunities for more integrated brine purification processes.

## Figures and Tables

**Table 2:** Different uses of extractants for magnesium removal.

Extractant	Type	Selectivity Mg^2+^	pH	Ref.
D2EHPA	Acidic organophosphate	High	2 to 4	[19]
Cyanex 272	Phosphinic acid	High	3 to 5	[49]
Oxalic acid	Organic chelating acid	Moderate	3 to 5.5	[50]
β-Diketone acids	Organic chelating	Moderate	5–6	[51]
EDTA	Hight chelating	High	5	[52]

**Table 3:** Comparison of extraction systems for Mg^2+^/Li^+^ separation under different aqueous-matrix and operating conditions.

Extractant System	Aqueous Matrix	pH	IL/Extractant Concentration [M]	Diluent	O/A Ratio (*v*/*v*)	Extraction Mg^2+^ (%)	Co-Extraction Li^+^ (%)	Mg/Li Ratio	Ref.
[A336][P204]	Synthetic model solution (50.42 g/L Mg^2+^)	6	0.8	MIBK	6:1	59.72	10.7	-	[68]
[A336][V10]	Synthetic model solution (54.04 g/L Mg^2+^)	7	0.8	MIBK	6:1	60.98	7.15	16.8	[56]
[HOEmin][NTf_2_] + C923	Synthetic model solution (0.10 M MgCl_2_)	-	0.2	p-cymene	1:1	100	-	-	[13]
[HOEmin][NTf_2_] + TBP	Synthetic model solution (0.10 M MgCl_2_)	-	0.2	p-cymene	1:1	75	-	-	[13]
HPMBP + Cyanex 923	Synthetic model solution (0.01 M MgCl_2_)	4.5	0.1	p-cymene	1:1	75	2.3	3.5	[51]
HTTA + Cyanex 923	Synthetic model solution (0.01 M MgCl_2_)	4.7	0.1	p-cymene	1:1	100	9.5	3.5	[51]
HDBM + Cyanex 923	Synthetic model solution (0.01 M MgCl_2_)	9.5	0.1	p-cymene	1:1	95	16	3.5	[51]

**Note:** The extraction efficiencies reported in this table were obtained under different experimental conditions and should not be interpreted as directly comparable performance values. Differences in aqueous-phase composition, Mg/Li ratio, pH, ionic-liquid concentration, diluent, O/A ratio, and extraction configuration can significantly influence Mg^2+^ extraction and Li^+^ co-extraction. Multistage or counter-current results are identified and discussed in the accompanying text where applicable.

## Data Availability

No new data were created or analyzed in this study. Data sharing is not applicable to this article
